# Particulate Matter and Associated Metals: A Link with Neurotoxicity and Mental Health

**DOI:** 10.3390/atmos12040425

**Published:** 2021-03-26

**Authors:** Nicole A. Potter, Gabriella Y. Meltzer, Oyemwenosa N. Avenbuan, Amna Raja, Judith T. Zelikoff

**Affiliations:** 1 Department of Environmental Medicine, New York University Grossman School of Medicine, 341 East 25th Street, New York, NY 10010, USA; 2 Department of Social and Behavioral Sciences, New York University School of Public Health, 715/719 Broadway, New York, NY 10003, USA

**Keywords:** particulate matter, air pollution, particulate air pollution, metal inhalation, ambient metals, particulate matter associated metals, neurotoxicity, mental health

## Abstract

Particulate air pollution (PM) is a mixture of heterogenous components from natural and anthropogenic sources and contributes to a variety of serious illnesses, including neurological and behavioral effects, as well as millions of premature deaths. Ultrafine (PM_0.1_) and fine-size ambient particles (PM_2.5_) can enter the circulatory system and cross the blood–brain barrier or enter through the optic nerve, and then upregulate inflammatory markers and increase reactive oxygen species (ROS) in the brain. Toxic and neurotoxic metals such as manganese (Mn), zinc (Zn), lead (Pb), copper (Cu), nickel (Ni), and barium (Ba) can adsorb to the PM surface and potentially contribute to the neurotoxic effects associated with PM exposure. Epidemiological studies have shown a negative relationship between exposure to PM-associated Mn and neurodevelopment amongst children, as well as impaired dexterity in the elderly. Inhaled PM-associated Cu has also been shown to impair motor performance and alter basal ganglia in schoolchildren. This paper provides a brief review of the epidemiological and toxicological studies published over the last five years concerning inhaled PM, PM-relevant metals, neurobiology, and mental health outcomes. Given the growing interest in mental health and the fact that 91% of the world’s population is considered to be exposed to unhealthy air, more research on PM and PM-associated metals and neurological health is needed for future policy decisions and strategic interventions to prevent public harm.

## 1. Introduction

Air pollution continues to pose a global environmental health risk that affects both the development and worsening of many health issues including cardiovascular disease, pulmonary illnesses, cancer, and central nervous system disorders [1]. According to the 2017 Global Burden of Disease Study, 4.9 million deaths and 1.4 billion disability-adjusted life years (DALYs) in 2017 were attributed to air pollution [1,2]. Previous mechanistic studies have reported that inhalation of air pollutants can provoke neuro-inflammation, oxidative stress, and dopaminergic neurotoxicity [2]. Particulate air pollution, composed mainly of organic and elemental carbon, metals, polycyclic aromatic hydrocarbons, inorganic compounds, nitrates, sulfates, and other organic materials (like polychlorinated biphenyls from industrial manufacturing), is a major component of air pollution and is of great research interest due to its well-documented associations with serious short- and long-term adverse health effects [1,3–7]. Particulate matter (PM) can be denoted by size and ranges from 10 μm to less than 0.1 μm in size. Fine PM particles (PM_2.5_) are between 2.5 and 0.1 μm in diameter, while ultrafine particles (UFP) are less than 0.1 μm in diameter (PM_0.1_) [6–9]. Major sources of PM include combustion of fossil fuels, traffic, and industrial and agricultural processes [1,4].

### 1.1. PM Distribution to the Brain

Particulate matter can enter the body through multiple routes of exposure including inhalation and ingestion [10]; the smaller the particle, the longer it can remain in the lungs [11]. Ultrafine particles in the lung act like gas molecules and can reach the circulatory system and potentially cross the blood–brain barrier [8,9,11–13]. Previous research studies also suggest that inhaled PM_2.5_ reaches the brain [11]. Fine and ultrafine PM can reach the brain through multiple pathways once it enters into the circulatory system [1,12]. Ultrafine particles (PM_0.1_) can pass through the blood–brain barrier, reach the vagal nerves, and enter the brain [1]. Alternatively, PM can bypass the blood–brain barrier by entering the brain via the olfactory bulb and fifth cranial nerve from direct inhalation through the nose [1,12]. The fact that fine and ultrafine PM can be transported directly through the olfactory pathway to the brain has been previously confirmed by the presence of particles in olfactory neurons and intracellular erythrocytes in the frontal lobe of the brain [12]. The presence of PM_2.5_ in the brain has been shown to cause cell cycle arrest, apoptosis of neurons, neuro-inflammation, induce oxidative stress and genetic damage leading to neurodegenerative changes and dopaminergic neurotoxicity, as well as structural damage to the myelin sheath [2,6,12,14–16].

### 1.2. PM Constituents

Particulate matter is a mixture of chemical compounds that can include inorganic and organic materials, as well as nitrates and sulfates [1,3,4,6]. The surface of ambient PM, particularly PM_2.5_, can carry viruses, bacteria, volatile organic compounds (VOCs), and heavy metals by adsorbing to the surface of the particle [6,7,10,13]. A number of studies have demonstrated high levels of adsorbed toxic heavy metals coming from both natural and anthropogenic sources that can adversely affect human health [3,10,11]. The types and concentrations of metals adsorbed to PM varies depending on the source and environment, but can range in concentration from 30 to 35 μg/m^3^ [17].

PM-associated heavy metals, including manganese (Mn), zinc (Zn), iron (Fe), cadmium (Cd), copper (Cu), arsenic (As), barium (Ba), lead (Pb), aluminum (Al), and nickel (Ni), are often significant metal components of PM_2.5_ [10,18,19]; previous studies have reported Mn, Ba, Ni, Fe, and Cu triggering inflammatory responses after exposure [4,18]. Maternal exposure during pregnancy to Mn, Ni, Pb, or Fe has previously been associated with increased risk of childhood autism, and exposure of pregnant mice to Mn has been linked with an acute inflammatory response resulting in neurotoxicity during fetal neurodevelopment [4,6,16,20]. Developmental Pb exposure has also been reported to be linked with schizophrenia due to antagonism of N-methyl-D-aspartate (NMDA) subtype of glutamate receptors (NMDAR); antagonism of NMDAR receptors can lead to decreased function, which could also play a role in the pathophysiology of schizophrenia [1]. Recent studies reported that the presence of PM and associated metals, Fe and Al, in the corpus callosum is associated with structural myelin sheath damage, which indicates that exposure to PM and associated heavy metals can disturb myelinogenesis [6,13,15,16].

### 1.3. PM Sources

Traffic emissions are a major source of heavy metal pollution in the ambient environment due to increased vehicle use and traffic congestion [9,19]. For example, automobile emissions release a number of heavy metals into the ambient environment including Cu, Zn, Cd, As, mercury (Hg), Mn, cobalt (Co), and Fe [10,21]. In addition, both Cu and Zn can be released into the air from tire abrasion, lubricants, and corrosion of vehicular parts, while Cd contamination arises from aging automobile tires, gasoline use, and car body and brake lining wear [19,21]. Industrial activity also contributes to particle-bound heavy metal air pollution, along with power plants, mining, metal smelting, and chemical plants [10,19]. Other sources of PM-associated heavy metal pollution originate from construction activity through building demolition and renovation, the spraying of pesticides and fungicides, as well as residential and commercial heating [10,19].

### 1.4. Air Pollution and Mental Health

Recently, studies have emerged that demonstrate the adverse effects of air pollution, either gases, particles, or a combination of both, on mental health [2]. The risk of psychosis has been linked to both genetic and environmental factors with increasing evidence that the environment can play a large part in influencing genetic effects through gene-environment interactions and epigenetic mechanisms [1]. Epidemiological studies reported that both short- and long-term exposure to PM_2.5_ were associated with greater odds of depression; the same relationship was not observed with inhalation of larger size particulate matter (PM_10_) [2]. There is also a reported relationship between urbanicity of birthplace/upbringing and a higher incidence of schizophrenia and other non-affective psychoses [1]. One of the speculated risk factors that could partially explain this association is exposure to particulate/gaseous air pollutants, which represents an underlying urbanicity risk factor [1]. Inhalation exposure to PM_2.5_ can also influence central nervous system development, resulting in an increased risk of later life depression, as well as influence cognitive disorders and create abnormalities in the architecture of brain white matter during childhood [7,12,20].

This contemporary review will focus on recent toxicological and epidemiological research over the last five years (2015–present) that considered the association between PM, particularly PM_2.5_, and selected soluble metals that have been shown in the literature to be associated with ambient PM and neurological/neurodevelopmental health outcomes. The toxicological studies reviewed herein focus on the association between fine and ultrafine PM exposure and neurological health effects. Review of these studies will help bring awareness to this well-deserved, but understudied, area of research, as well as reveal research gaps that need to be addressed to protect highly exposed individuals.

## 2. Methods

Review of the literature for this review took place during May–June 2020 and reputable databases including PubMed, Google Scholar, and Web of Science, were used to search for relevant literature. Search terms included various combinations of the following key words and phrases: “particulate matter”, “PM”, “particulate matter metals”, “ambient metals”, “brain”, “neurotoxicity”, “mental health”, “air pollution”, “metal inhalation”, “nanoparticles”, “heavy metal neurotoxicity in vivo”, “heavy metal neurotoxicity in vitro”, “PM_2.5_ neurotoxicity in animal models”, “PM_2.5_ and oxidative stress in mouse brains”, and “inhaled PM_2.5_ and neurotoxicity”. Specific heavy metals that have been reported to be associated with particulate matter and neurotoxicity were also added to searches including (but, not limited to) “cadmium”, “nickel”, “zinc”, “manganese”, “copper”, and “lead”. Eligibility criteria involved recent publication year, particularly publications within the last five years. Toxicological and epidemiological studies that focused particularly on fine and ultra-fine PM and associated ambient metals, were preferred, as well as full-text publications. Geographical location of studies was not taken into account. Exclusion criteria included the study year of publication, studies that were not fully available online, and studies examining general air pollution exposure without going into detail of constituents.

Rationale for this review paper is to assess recent research examining the association between inhalation exposure to PM and associated metals and brain health.

## 3. PM and Mental Health: Epidemiological Studies

A large body of epidemiological literature has recently explored the association between exposure to airborne PM and adverse neurological outcomes [22–26]. Other investigators have studied neurological outcomes based on exposure to heavy metals, but without a specified exposure route [27–32]. However, only a select few studies have examined the relationship between inhalation exposure to PM component metals and neurological health. Given the link emerging between inhaled PM and its metal constituents and neurological health, this section will review recent information in this area to help delineate research pathways (Table 1).

Several researchers have studied the association between airborne Mn exposure and neurodevelopmental outcomes among children [33]. A 2018 cross-sectional study found a significant negative association between airborne Mn exposure and Full Scale IQ (β = −1.91, *p* < 0.05; β and *p* refer to the regression coefficient and statistical significance, respectively) among 106 children ages seven to nine living in East Liverpool, Ohio, which hosts both a hazardous waste incinerator and a Mn processor [34]. These results build upon the authors’ earlier study in 2015 examining children ages seven to nine in Cambridge and Marietta, Ohio, which houses the longest operating ferromanganese refinery in the US [35]. In another cross-sectional study of 225 children ages seven to twelve in Simões Filho, Brazil, the association between exposure to both Mn and Pb in dust and intellectual deficiencies was examined [36]. The authors not only showed that an 8.6 point drop in IQ was significantly associated with a ten-fold increase in blood Pb levels (BLL), but also that this association was increased by 27.9% among those children with high levels of toenail Mn (β = *−*8.70, *p* = 0.036). These studies jointly demonstrate that childhood exposure to Mn and/or Pb, associated with inhaled PM emitted from industrial sources, is associated with reduced cognitive function and intellectual abilities.

At the other end of the life course, Pesch et al. (2017) studied occupational exposure to Mn and fine motor skills among 1232 former male welders or steel workers in the German cities of Buchom, Essen, and Mülheim. The researchers demonstrated that cumulative Mn inhalation exposure was associated with impaired dexterity measured by errors in line tracing, steadiness, and/or aiming and tapping hits [37]. Contrary to other researchers’ findings, a 2019 study by Palzes et al. that examined 48 farmworkers in Zarcero County, Puerto Rico found no association between inhalation exposure to Mn-associated ethylene bisdithiocarbamate fungicide and working memory brain activity [38]. These differences could be due to the small sample size in the Palzes et al. study. Further research is needed to better understand the effects of airborne Mn exposure on older adults’ neurological health, especially those with extended occupational exposure.

Airborne exposure to PM-associated metals other than Mn or Pb have also been shown to be associated with poor neurological outcomes. For example, Pujol et al. (2016) examined the association between indoor and outdoor exposure to airborne Cu in Barcelona schools and impaired motor performance and altered basal ganglia in children ages eight through twelve. The authors demonstrated that greater ambient Cu exposure was significantly associated with both poorer motor performance based on speed (β = 2.2, *p* = 0.006) and consistency (β = 2.9, *p* < 0.00001) of reaction times, as well as altered basal ganglia structures [39].

Two recent studies, one by Lubczyńska et al. (2020) and the other by Liu et al. (2018) examined inhaled ambient metals and neurological health in the context of exposure to PM. A 2020 longitudinal study by Lubczyńska et al. of 2954 children in the Netherlands evaluated inhalation exposure of PM at different sizes and its components throughout pregnancy and childhood, as well as their association with white matter microstructure in pre-adolescents. The authors reported that a 5 μg/m^3^ increase in PM_2.5_ exposure during pregnancy was significantly associated with a −0.71 unit decrease in children’s fractional anisotropy, a measure of connectivity in the brain (*p* < 0.05). A 100 ng/m^3^ increase in prenatal elemental silicon exposure was also significantly associated with a 0.06 unit increase in mean diffusivity (*p* < 0.05). In addition, a 10 ng/m^3^ increase in Zn exposure during childhood was significantly associated with a 0.03 unit increase in mean diffusivity (*p* < 0.05). Both low fractional anisotropy and high mean diffusivity are indicative of unhealthy brain development and potential psychiatric disorders [24]. A randomized crossover trial conducted by Liu et al. (2018), of 53 healthy, non-smoking Canadian men and women exposed to urban coarse (>PM_10_) or concentrated ambient PM_0.1_, demonstrated that ubiquitin

C-terminal hydrolase L1 (UCHL1), a biomarker for traumatic brain injury, increased 11% (95% CI: 1.2%, 21%) and 14% (95% CI: 0.3%, 29%) one and twenty-one hours post-exposure to Ba, respectively. The authors also reported that vanillylmandelic acid, a urinary neural biomarker often used to diagnose neuroblastoma, increased 29% (95% CI: 3%, 54%) one-hour post-exposure to Al [18]. These findings on anatomic brain alterations support other results demonstrating associations between inhaled metal exposure and adverse neurological outcomes [18,24]. However, whether exposure to these heavy metals that are known to have neurological effects continue to have similar effects when they become associated with PM, still requires further research.

In conclusion, the reviewed epidemiological studies have revealed: links between ambient metal exposure and intellectual deficiencies; declines in motor performance; alterations in brain development; and changes in specific neural biomarkers associated with brain injury. However, major gaps still exist in understanding the relationship between neurotoxicity and exposure to ambient PM and its associated metals, as very few epidemiological studies focus on inhaled ambient metal exposure associated with PM.

## 4. PM and Mental Health: Toxicology Studies

The paucity of research addressing the toxicological and molecular mechanisms of inhaled heavy metals associated with PM and their correlation to brain health makes it difficult to reach definitive conclusions. Much of the research on inhaled heavy metals, PM, and health effects on the brain focuses primarily on epidemiological associations and less on potential causative mechanisms. One issue in this regard is the difficulty involved in separating specific constituents from a heterogenous mixture of compounds to investigate causal relationships. This section will cover a number of recent toxicological studies (2015 to present) in order to attempt to delineate the association between PM exposure and neurotoxicity (Table 2).

Many studies that address the impact of inhaled PM on the brain and/or mental health use animal models. In an investigation by Ning et al. (2018), mice at different developmental stages (four weeks, four months, or ten months of age) were exposed to PM_2.5_ (3 mg/kg) by inhalation every other day for a total of four weeks to examine potential deterioration of spatial learning and memory directly or indirectly related to the hippocampal metabolic region. The findings from this study demonstrate that exposed four-week-old mice had impaired spatial and learning memory [40]. The investigators also examined metabolic changes related to energy, cholesterol, and aspartic acid in hippocampal tissues of the juvenile mice [40]. Additionally, inhalation of PM_2.5_ has recently been suggested to be linked with gut microbial alterations, leading to worsening of Alzheimer’s disease (AD) in AD-prone mice [41]. To test this hypothesis, Fu et al. (2020) examined the relationship between inhaled PM_2.5_-induced microbial gut alterations and AD. After eight weeks of exposure to PM_2.5_ at 61 μg/m^3^ (which represented 80% of the atmospheric concentration of 87 μg/m^3^) AD-mice had increased pro-inflammatory cytokine concentrations (i.e., tumor necrosis factor-alpha [TNF-a] and interleukin [IL-6]) in the brain and intestines alongside altered microbial diversity in the intestine and feces compared to two control groups (AD-mice and B6 mice exposed to filtered air) [41]. Coupled with other previous studies, Fu et al. (2020) concluded that inhalation exposure to PM_2.5_ could intensify inflammation in the brain and intestines, as well as alter microbial diversity in mice with ongoing AD.

Studies have shown that inhalation of PM upregulates inflammatory markers in certain brain regions. As inflammation is a major mechanism underlying neurodegenerative diseases [1,2,7,8,12,14,40–44], it is worthwhile to address the mechanisms behind the disease etiology. Researchers have assessed the activation of NF-κB—an important transcription factor in pro-inflammatory responses—and compared it to lipid peroxidation and nitric oxide (NO) production in cell-based systems [42]. In a study conducted by Zhang et al. (2019), exposure to urban-based ambient PM produced alterations in NF-κB activation. Of the three biological responses assessed in this study, NF-κB activation in vitro was most highly correlated with in vivo neurotoxic responses, whereas lipid peroxidation and NO production had little correlation in vitro to in vivo neurotoxic responses [42]. These studies suggest that NF-κB may be linked to PM-induced neurotoxicity in vivo.

A study by Liu et al. (2019) addressed PM_2.5_-induced development of neurodegenerative diseases, as well as potential mechanisms underlying the observed effects. Results of this inhalation study demonstrated that exposed mice had a reduction in escape latency (EL) after five days, as well as the presence of protein aggregates in the cerebral cortex of exposed mice and neuronal accumulation of amyloid beta (Aβ)_1–42_ [43]. There was also a significant increase in the levels of reactive oxygen species (ROS) seen in all groups of exposed mice compared to the control [43]. Moreover, exposure to 19.3 mg PM_2.5_/kg/day dramatically increased malondialdehyde (MDA) levels in the mouse brain [43]. Conversely, brain-associated glutathione (GSH) content in mice exposed to either 1.93 mg PM_2.5_/kg/day or 19.3 mg PM_2.5_/kg/day decreased significantly [43]. A decrease was also observed in brain-associated superoxide dismutase (SOD) levels [43]. Moreover, gene expression of NF-κB, TNF-α, and IL-1β increased significantly in mice exposed to 19.3 mg PM_2.5_/kg/day compared to the control group [43]. Inhalation exposure of PM_2.5_ has also been reported to alter brain inflammation, which appears to enhance AD progression mainly through β-secretase 1 (BACE1) activation catalyzed by β-site amyloid precursor protein (APP) [44]. In a study by Ku et al. (2017), mice received PM_2.5_ via oropharyngeal aspiration at doses of either 1 or 5 mg/kg every other day for four weeks to address the hypothesis that PM inhalation promotes BACE1 and γ-secretase leading to antibody synthesis and, in turn, synaptic and cognitive impairment [44]. Results of this study demonstrated that PM_2.5_-induced expressions of APP and BACE1 increased following pulmonary aspiration of PM_2.5_ at 5 mg/kg compared to aspiration of PM_2.5_ at a five-fold lower dose (1 mg/kg) [44]. The aforementioned results again show a positive relationship between AD progression and inhalation of PM_2.5_.

In a separate study, Li et al. (2018) explored the effects that PM_2.5_ had on neurotoxicity and behavioral and neurotransmitter changes in rats. Sprague–Dawley male rats were randomly assigned to different PM dose groups including 0 (control), 5 mg/kg, 10 mg/kg or 20 mg/kg per week and exposed for up to 12 weeks by tracheal perfusion [45]. This study revealed that rats treated with 10 mg/kg PM_2.5_/week and 20 mg/kg/week had significantly higher levels of hippocampal Mn tissue content compared to those age-matched rats in the control group [45]. Additionally, this study demonstrated that exposure to PM_2.5_ decreased spatial cognition, as well as exploratory behavior abilities at a significant rate compared to control rats [45]. Likewise, in another study, perinatal exposure of mice to concentrated PM_2.5_ (135.8 ± 13.2 5 μg/m^3^) was related to an autism spectrum disorder (ASD)-like phenotype in a sex-dependent manner; male mice had more severe behavioral deficits compared to exposure-matched female mice [4]. Taken together, these rodent studies suggest a correlation between PM_2.5_ exposure and neurocognitive alterations.

Taken together, these toxicological studies demonstrate that, ambient exposure to PM_2.5_ has been linked to: deterioration in cognitive function; changes in brain metabolism; and an increase in neural inflammation in animal models. However, more research investigating the underlying mechanisms explaining the relationship between PM and their associated metal constituents and neurotoxicity, is needed.

## 5. Conclusions

In 2018, the World Health Organization (WHO) considered 91% of the world’s population to be exposed to unhealthy air (PM_2.5_ air quality guideline value for the PM2.5 annual average concentration is 10 μg/m^3^) [46]. By 2050, 68% of the world’s population is expected to be living in urban areas, and may therefore be exposed continuously to unhealthy air pollution levels [14]. As particulate air pollution continues to pose health risks throughout the world [46], and published studies in both humans and animal models have demonstrated a negative association between airborne metal inhalation and brain health, including neurodevelopmental outcomes, motor performance, spatial learning and memory, and alterations in brain structure and chemistry, more research studies are needed in this relatively unexplored scientific field. In addition, children and fetuses appear to be a particularly susceptible population to these exposures as their brains are still developing [4,6,13,15,25].

Mental health disorders such as dementia, depression, schizophrenia, and other forms of psychoses are critical health issues that need to be better understood, particularly given the large number of individuals throughout the world who suffer from such illnesses. Research that aids in a better understanding of potential risk factors, such as particulate air pollution and inhaled metals that contribute to neurological dysfunction and impaired mental health is critical for developing and administering appropriate treatments. This is especially true as neurodegenerative diseases increase as the population ages and life expectancies are extended [14]. The number of people predicted to live with dementia in the next 30 years is expected to triple and there are still no available curative treatments [14]. Thus, to better understand how particulate air pollution exposure and its constituents, in particular, are involved in the pathophysiology of brain health and various mental health disorders, scientists and policy-makers need to step up to confirm this understudied area of research as a priority to protect human health.

## 6. Gaps in Literature and Future Directions

The intersection of inhaled ambient PM and PM-associated metals with neurobiology and mental health is a relatively new field. As such, there are many untapped areas of research, including the molecular and cellular mechanisms underlying PM and the PM-associated metal-induced development and escalation of neurological diseases. Most epidemiological and toxicological investigations in this area focus on the effects of PM in its entirety, rather than explore the relationship between PM-associated constituents and neurological health.

Looking at the present and towards the future, another source of exposure to PM-associated metals are electronic cigarette aerosols. However, except for a recent paper by Church et al. (2020), data on the effects of e-cigarette aerosol-associated metals on mental health outcomes are extremely limited. Another important source of PM-associated metals to consider is the use of metal-based nanoparticles in a variety of industries and medicines, which could also lead to important brain health issues in the future [47–49]. With limited knowledge of neurobiology and mental health effects of inhaled metals via particle pollution, it is difficult to adequately protect vulnerable populations such as children, pregnant women, marginalized populations, and the elderly from additional health risks not previously recognized.

## Figures and Tables

**Table 1.** Summary of epidemiological studies on particulate matter (PM) and associated metals and their effects on brain health.

Study	Study Design	Inhalation Exposure	Results
Haynes et al., 2018	Expansion of Communities Actively Researching Exposure (CARES) cohort (*n* = 106; East Liverpool Ohio, US)	Mn	Exposure negatively associated with Full Scale IQ measurements among children
Haynes et al., 2015	Expansion of CARES cohort (*n* = 404; East Liverpool Ohio, US)	Mn	Exposure negatively associated with Full Scale IQ measurements among children
Menezes-Filho et al., 2018	Cross-sectional study (*n* = 225; Simões Filho, Bahia, Brazil)	Mn and Pb	Exposure associated with intellectual deficiencies (IQ)
Pesch et al., 2017	Second follow up survey of Heinz Nixdorf Recall Study (HNRS) cohort (*n* = 1232; Germany)	Mn	Exposure associated with impaired dexterity
Palzes et al., 2019	Cross-sectional (*n* = 48; Zarcero County, Costa Rica)	Mn-associated fungicide	Exposure not associated with changes in working memory
Pujol et al., 2016	BREATHE Project cohort (*n* = 2836; Barcelona, Spain)	Cu	Exposure associated with impaired motor performance and altered basal ganglia in children
Lubczyńska et al., 2020	Generation R cohort (*n* = 2954; Rotterdam, Netherlands)	Various sizes of PM (including PM_2.5_) and metal constituents (including Si and Zn)	PM_2.5_ exposure associated with decrease in children’s fractional anisotropy Si exposure associated with increase in mean diffusivity Zn exposure associated with increase in mean diffusivity
Liu et al., 2018	Single-blind randomized cross-over trial (*n* = 53; Toronto, Canada)	Urban coarse ambient PM (>PM_10_); Concentrated ambient PM (PM_0.1_); Related metals (Al, Ba)	Ba exposure associated with increase in ubiquitin C-terminal hydrolase L1 Al exposure associated with increased urinary neural marker vanillylmandelic acid

**Table 2.** Summary of toxicological studies on particulate matter (PM_2.5_) and associated metals and their effects on brain health.

Study	Research Model	Route of Exposure	PM_2.5_ Treatments	Results
Ning et al., 2018	C57BL/6 mice at 4 wk, 4 mo, 10 mo (*n* = 8–10 mice/group)	Oropharyngeal aspiration	3 mg/kg every other day for 4 wk; saline (control)	Exposed mice showed deterioration of spatial learning and memory
Fu et al., 2020	APP/PS1 double transgenic AD-mice; littermate B6 mice (*n* = 5 mice/group)	Inhalation via exposure chambers	61 μg/kg for 8 wk; filtered air (control)	Exposed mice with AD showed increase in inflammation in brain and intestines
Zhang et al., 2019	in vitro: THP1- Blue NF-κB human monocytes; BV-2 cell lines (*n* = 4) in vivo: C57BL/6NJ male mice (*n* = 10 mice/group)	in vitro: PM treatment in vivo: inhalation nebulizers	in vivo: re-aerosolized exposure for 3- or 8-wks; filtered air (control)	Exposure produced alterations in NF-κB
Liu et al., 2019	C57BL/6J male mice (n = 10 mice/group)	Intranasal instillation	Two rounds of exposure to various concentrations daily for 7 days (vitamin E added to second round); filtered air (control)	Exposed mice experienced reduction in escape latency; presence of protein aggregates in cerebral cortex; neuronal accumulation of amyloid beta1–42; increase in reactive oxygen species (ROS); increase in brain levels of malondialdehyde; decrease in
Ku et al., 2017	C57BL/6 male mice (*n* = 13–14 mice/group)	Oropharyngeal aspiration	1 and 5 mg/kg every other day for 4-wks; saline (control)	brain-associated glutathione content and superoxide dismutase; increase in NF-κB, IL-1β and TNF-α Exposed mice experienced induced expression of amyloid precursor protein and β-secretase 1
Li et al., 2018	Sprague-Dawley male rats (*n* = 8 rats/group)	Tracheal perfusion of solutions	5,10, 20 mL/kg solution once a week for up to 12-wks; saline (control)	Exposed mice experienced elevated levels of manganese in hippocampal tissue compared to control; decrease in spatial cognition and exploratory behaviors
Church et al., 2018	B6C3F1 pregnant female mice and offspring (*n* = 26 control mice; *n* = 31 exposure mice)	Inhalation via exposure chamber	Peri-natal (135.8 μg/m^3^; 6 hr/d 7 d/wk) post-natal (135.8 μg/m^3^; 2 hr/d 7 d/wk); filtered air (control)	Exposed mice exhibited an autism spectrum-like phenotype in a sex-dependent manner

## References

[1] Attademo L; Bernardini F; Garinella R; Compton MT Environmental pollution and risk of psychotic disorders: A review of the science to date. Schizophr. Res 2017, 181, 55–59. 27720315 10.1016/j.schres.2016.10.003

[2] Fan SJ; Heinrich J; Bloom MS; Zhao TY; Shi TX; Feng WR; Sun Y; Shen JC; Yang ZC; Yang BY; Ambient air pollution and depression: A systematic review with meta-analysis up to 2019. Sci. Total Environ 2020, 701, 134721. 31715478 10.1016/j.scitotenv.2019.134721

[3] Hamanaka RB; Mutlu GM Particulate Matter Air Pollution: Effects on the Cardiovascular System. Front. Endocrinol 2018, 9, 680. 10.3389/fendo.2018.00680 PMC6250783 30505291

[4] Church JS; Tijerina PB; Emerson FJ; Coburn MA; Blum JL; Zelikoff JT; Schwartzer JJ Perinatal exposure to concentrated ambient particulates results in autism-like behavioral deficits in adult mice. Neurotoxicology 2018, 65, 231–240. 29104007 10.1016/j.neuro.2017.10.007 PMC5857220

[5] Yang Y; Ruan Z; Wang X; Yang Y; Mason TG; Lin H; Tian L Short-term and long-term exposures to fine particulate matter constituents and health: A systematic review and meta-analysis. Environ. Pollut 2019, 247, 874–882. 30731313 10.1016/j.envpol.2018.12.060

[6] Klocke C; Sherina V; Graham UM; Gunderson J; Allen JL; Sobolewski M; Blum JL; Zelikoff JT; Cory-Slechta DA Enhanced cerebellar myelination with concomitant iron elevation and ultrastructural irregularities following prenatal exposure to ambient particulate matter in the mouse. Inhal. Toxicol 2018, 30, 381–396. 30572762 10.1080/08958378.2018.1533053 PMC6400059

[7] Allen JL; Klocke C; Morris-Schaffer K; Conrad K; Sobolewski M; Cory-Slechta DA Cognitive Effects of Air Pollution Exposures and Potential Mechanistic Underpinnings. Curr. Environ. Health Rep 2017, 4, 180–191. 28435996 10.1007/s40572-017-0134-3 PMC5499513

[8] Jew K; Herr D; Wong C; Kennell A; Morris-Schaffer K; Oberdorster G; O’Banion MK; Cory-Slechta DA; Elder A Selective memory and behavioral alterations after ambient ultrafine particulate matter exposure in aged 3xTgAD Alzheimer’s disease mice. Part. Fibre Toxicol 2019, 16, 45. 31771615 10.1186/s12989-019-0323-3 PMC6878709

[9] Morris-Schaffer K; Merrill A; Jew K; Wong C; Conrad K; Harvey K; Marvin E; Sobolewski M; Oberdorster G; Elder A; Effects of neonatal inhalation exposure to ultrafine carbon particles on pathology and behavioral outcomes in C57BL/6J mice. Part. Fibre Toxicol 2019, 16, 10. 30777081 10.1186/s12989-019-0293-5 PMC6379948

[10] Liu Y; Li S; Sun C; Qi M; Yu X; Zhao W; Li X Pollution Level and Health Risk Assessment of PM2.5-Bound Metals in Baoding City Before and After the Heating Period. Int. J. Environ. Res. Public Health 2018, 15, 2286. 30340357 10.3390/ijerph15102286 PMC6210169

[11] Kim EA Particulate Matter (Fine Particle) and Urologic Diseases. Int. Neurourol. J 2017, 21, 155–162. 28954465 10.5213/inj.1734954.477 PMC5636961

[12] Gladka A; Rymaszewska J; Zatonski T Impact of air pollution on depression and suicide. Int. J. Occup. Med. Environ. Health 2018, 31, 711–721. 30281038 10.13075/ijomeh.1896.01277

[13] Klocke C; Allen JL; Sobolewski M; Mayer-Proschel M; Blum JL; Lauterstein D; Zelikoff JT; Cory-Slechta DA Neuropathological Consequences of Gestational Exposure to Concentrated Ambient Fine and Ultrafine Particles in the Mouse. Toxicol. Sci 2017, 156, 492–508. 28087836 10.1093/toxsci/kfx010 PMC6074840

[14] Grande G; Ljungman PLS; Eneroth K; Bellander T; Rizzuto D Association Between Cardiovascular Disease and Long-term Exposure to Air Pollution With the Risk of Dementia. JAMA Neurol. 2020, 77, 801–809. 32227140 10.1001/jamaneurol.2019.4914 PMC7105952

[15] Klocke C; Allen JL; Sobolewski M; Blum JL; Zelikoff JT; Cory-Slechta DA Exposure to fine and ultrafine particulate matter during gestation alters postnatal oligodendrocyte maturation, proliferation capacity, and myelination. Neurotoxicology 2018, 65, 196–206. 29079486 10.1016/j.neuro.2017.10.004 PMC5857223

[16] Cory-Slechta DA; Sobolewski M; Marvin E; Conrad K; Merrill A; Anderson T; Jackson BP; Oberdorster G The Impact of Inhaled Ambient Ultrafine Particulate Matter on Developing Brain: Potential Importance of Elemental Contaminants. Toxicol. Pathol 2019, 47, 976–992. 31610749 10.1177/0192623319878400 PMC6911038

[17] Popoola LT; Adebanjo SA; Adeoye BK Assessment of atmospheric particulate matter and heavy metals: A critical review. Int. J. Environ. Sci. Technol 2017, 15, 935–948.

[18] Liu L; Urch B; Szyszkowicz M; Evans G; Speck M; Van Huang A; Leingartner K; Shutt RH; Pelletier G; Gold DR; Metals and oxidative potential in urban particulate matter influence systemic inflammatory and neural biomarkers: A controlled exposure study. Environ. Int 2018, 121, 1331–1340. 30420132 10.1016/j.envint.2018.10.055 PMC6396878

[19] Hou S; Zheng N; Tang L; Ji X; Li Y; Hua X Pollution characteristics, sources, and health risk assessment of human exposure to Cu, Zn, Cd and Pb pollution in urban street dust across China between 2009 and 2018. Environ. Int 2019, 128, 430–437. 31082721 10.1016/j.envint.2019.04.046

[20] Cory-Slechta DA; Allen JL; Conrad K; Marvin E; Sobolewski M Developmental exposure to low level ambient ultrafine particle air pollution and cognitive dysfunction. Neurotoxicology 2018, 69, 217–231. 29247674 10.1016/j.neuro.2017.12.003 PMC5999548

[21] Li N; Han W; Tang J; Bian J; Sun S; Song T Pollution Characteristics and Human Health Risks of Elements in Road Dust in Changchun, China. Int. J. Environ. Res. Public Health 2018, 15, 1843. 30150528 10.3390/ijerph15091843 PMC6164438

[22] Braithwaite I; Zhang S; Kirkbride JB; Osborn DPJ; Hayes JF Air Pollution (Particulate Matter) Exposure and Associations with Depression, Anxiety, Bipolar, Psychosis and Suicide Risk: A Systematic Review and Meta-Analysis. Environ. Health Perspect 2019, 127, 126002. 31850801 10.1289/EHP4595 PMC6957283

[23] Liu L; Urch B; Szyszkowicz M; Speck M; Leingartner K; Shutt R; Pelletier G; Gold DR; Scott JA; Brook JR; Influence of exposure to coarse, fine and ultrafine urban particulate matter and their biological constituents on neural biomarkers in a randomized controlled crossover study. Environ. Int 2017, 101, 89–95. 28117141 10.1016/j.envint.2017.01.010 PMC5348252

[24] Lubczyńska MJ; Muetzel RL; El Marroun H; Basagana X; Strak M; Denault W; Jaddoe VWV; Hillegers M; Vernooij MW; Hoek G; Exposure to Air Pollution during Pregnancy and Childhood, and White Matter Microstructure in Preadolescents. Environ. Health Perspect 2020, 128, 27005. 32074458 10.1289/EHP4709 PMC7064320

[25] Mortamais M; Pujol J; Martinez-Vilavella G; Fenoll R; Reynes C; Sabatier R; Rivas I; Forns J; Vilor-Tejedor N; Alemany S; Effects of prenatal exposure to particulate matter air pollution on corpus callosum and behavioral problems in children. Environ. Res 2019, 178, 108734. 31539824 10.1016/j.envres.2019.108734 PMC6892268

[26] Rivas I; Basagana X; Cirach M; Lopez-Vicente M; Suades-Gonzalez E; Garcia-Esteban R; Alvarez-Pedrerol M; Dadvand P; Sunyer J Association between Early Life Exposure to Air Pollution and Working Memory and Attention. Environ. Health Perspect 2019, 127, 57002. 31070940 10.1289/EHP3169 PMC6791117

[27] Carroll CR; Noonan C; Garroutte EM; Navas-Acien A; Verney SP; Buchwald D Low-level inorganic arsenic exposure and neuropsychological functioning in American Indian elders. Environ. Res 2017, 156, 74–79. 28334644 10.1016/j.envres.2017.03.018 PMC5485900

[28] Dickerson AS; Rotem RS; Christian MA; Nguyen VT; Specht AJ Potential Sex Differences Relative to Autism Spectrum Disorder and Metals. Curr. Environ. Health Rep 2017, 4, 405–414. 28988324 10.1007/s40572-017-0164-x PMC6508872

[29] Guo J; Wu C; Zhang J; Qi X; Lv S; Jiang S; Zhou T; Lu D; Feng C; Chang X; Prenatal exposure to mixture of heavy metals, pesticides and phenols and IQ in children at 7 years of age: The SMBCS study. Environ. Int 2020, 139, 105692. 32251899 10.1016/j.envint.2020.105692

[30] Leonhard MJ; Chang ET; Loccisano AE; Garry MR A systematic literature review of epidemiologic studies of developmental manganese exposure and neurodevelopmental outcomes. Toxicology 2019, 420, 46–65. 30928475 10.1016/j.tox.2019.03.004

[31] Park JH; Seo JH; Hong YS; Kim YM; Kang JW; Yoo JH; Chueh HW; Lee JH; Kwak MJ; Kim J; Blood lead concentrations and attention deficit hyperactivity disorder in Korean children: A hospital-based case control study. BMC Pediatr. 2016, 16, 156. 27659349 10.1186/s12887-016-0696-5 PMC5034496

[32] Min JY; Min KB Blood cadmium levels and Alzheimer’s disease mortality risk in older US adults. Environ. Health 2016, 15, 69. 27301955 10.1186/s12940-016-0155-7 PMC4908725

[33] Liu W; Xin Y; Li Q; Shang Y; Ping Z; Min J; Cahill CM; Rogers JT; Wang F Biomarkers of environmental manganese exposure and associations with childhood neurodevelopment: A systematic review and meta-analysis. Environ. Health 2020, 19, 104. 33008482 10.1186/s12940-020-00659-x PMC7531154

[34] Haynes EN; Sucharew H; Hilbert TJ; Kuhnell P; Spencer A; Newman NC; Burns R; Wright R; Parsons PJ; Dietrich KN Impact of air manganese on child neurodevelopment in East Liverpool, Ohio. Neurotoxicology 2018, 64, 94–102. 28888663 10.1016/j.neuro.2017.09.001 PMC5809274

[35] Haynes EN; Sucharew H; Kuhnell P; Alden J; Barnas M; Wright RO; Parsons PJ; Aldous KM; Praamsma ML; Beidler C; Manganese Exposure and Neurocognitive Outcomes in Rural School-Age Children: The Communities Actively Researching Exposure Study (Ohio, USA). Environ. Health Perspect 2015, 123, 1066–1071. 25902278 10.1289/ehp.1408993 PMC4590758

[36] Menezes-Filho JA; Carvalho CF; Rodrigues JLG; Araujo CFS; Dos Santos NR; Lima CS; Bandeira MJ; Marques BLS; Anjos ALS; Bah HAF; Environmental Co-Exposure to Lead and Manganese and Intellectual Deficit in School-Aged Children. Int. J. Environ. Res. Public Health 2018, 15, 2418. 30384464 10.3390/ijerph15112418 PMC6266231

[37] Pesch B; Casjens S; Weiss T; Kendzia B; Arendt M; Eisele L; Behrens T; Ulrich N; Pundt N; Marr A; Occupational Exposure to Manganese and Fine Motor Skills in Elderly Men: Results from the Heinz Nixdorf Recall Study. Ann. Work Expo. Health 2017, 61, 1118–1131. 29136419 10.1093/annweh/wxx076 PMC6824529

[38] Palzes VA; Sagiv SK; Baker JM; Rojas-Valverde D; Gutierrez-Vargas R; Winkler MS; Fuhrimann S; Staudacher P; Menezes-Filho JA; Reiss AL; Manganese exposure and working memory-related brain activity in smallholder farmworkers in Costa Rica: Results from a pilot study. Environ. Res 2019, 173, 539–548. 30991177 10.1016/j.envres.2019.04.006 PMC6581040

[39] Pujol J; Fenoll R; Macia D; Martinez-Vilavella G; Alvarez-Pedrerol M; Rivas I; Forns J; Deus J; Blanco-Hinojo L; Querol X; Airborne copper exposure in school environments associated with poorer motor performance and altered basal ganglia. Brain Behav. 2016, 6, e00467. 27134768 10.1002/brb3.467 PMC4842931

[40] Ning X; Li B; Ku T; Guo L; Li G; Sang N Comprehensive hippocampal metabolite responses to PM_2.5_ in young mice. Ecotoxicol. Environ. Saf 2018, 165, 36–43. 30179763 10.1016/j.ecoenv.2018.08.080

[41] Fu P; Bai L; Cai Z; Li R; Yung KKL Fine particulate matter aggravates intestinal and brain injury and affects bacterial community structure of intestine and feces in Alzheimer’s disease transgenic mice. Ecotoxicol. Environ. Saf 2020, 192, 110325. 32078839 10.1016/j.ecoenv.2020.110325

[42] Zhang H; Haghani A; Mousavi AH; Cacciottolo M; D’Agostino C; Safi N; Sowlat MH; Sioutas C; Morgan TE; Finch CE; Cell-based assays that predict in vivo neurotoxicity of urban ambient nano-sized particulate matter. Free Radic. Biol. Med 2019, 145, 33–41. 31542466 10.1016/j.freeradbiomed.2019.09.016 PMC7207020

[43] Liu X; Zhang Y; Yang X PM2.5 induced neurodegenerative-like changes in mice and the antagonistic effects of vitamin E. Toxicol. Res 2019, 8, 172. 10.1039/c8tx00333e PMC6425994 30997020

[44] Ku T; Li B; Gao R; Zhang Y; Yan W; Ji X; Li G; Sang N NF-kappaB-regulated microRNA-574–5p underlies synaptic and cognitive impairment in response to atmospheric PM2.5 aspiration. Part. Fibre Toxicol 2017, 14, 34. 28851397 10.1186/s12989-017-0215-3 PMC5575838

[45] Li Q; Zheng J; Xu S; Zhang J; Cao Y; Qin Z; Liu X; Jiang C The neurotoxicity induced by PM2.5 might be strongly related to changes of the hippocampal tissue structure and neurotransmitter levels. Toxicol. Res 2018, 7, 1144–1152. 10.1039/c8tx00093j PMC6220725 30510684

[46] Ambient (Outdoor) Air Pollution. Available online: https://www.who.int/news-room/fact-sheets/detail/ambient-(outdoor)-air-quality-and-health (accessed on 8 February 2021).

[47] Anastopoulos I; Hosseini-Bandegharaei A; Fu J; Mitropoulos AC; Kyzas GZ Use of nanoparticles for dye adsorption: Review. J. Dispers. Sci. Technol 2017, 39, 836–847.

[48] Ko S; Huh C Use of nanoparticles for oil production applications. J. Petrol. Sci. Eng 2019, 172, 97–114.

[49] Blum JL; Xiong JQ; Hoffman C; Zelikoff JT Cadmium associated with inhaled cadmium oxide nanoparticles impacts fetal and neonatal development and growth. Toxicol. Sci 2012, 126, 478–486. 22240978 10.1093/toxsci/kfs008 PMC3307609

